# Supplementary material for: Evaluation of Pharmacokinetics and Pharmacodynamics of Deferasirox in Pediatric Patients
Source: Pharmaceutics. 2021 Aug 11;13(8):1238. doi: 10.3390/pharmaceutics13081238 (PMC8401444; doi:10.3390/pharmaceutics13081238)
Supplement: Supplementary file 1 [file pharmaceutics-13-01238-s001.zip › pharmaceutics-1310929-supplementary.pdf]

pharmaceutics

MDPI

Supplementary Materials: Evaluation of Pharmacokinetics and Pharmacodynamics of Deferasirox in Pediatric Patients

Laura Galeotti, Francesco Ceccherini, Carmen Fucile, Valeria Marini, Antonello Di Paolo, Natalia Maximova and Francesca Mattioli

Table S1. Predicted and measured C_{trough} for in the present patient population and statistical analysis showing significant differences among groups with and without hepatic/renal adverse events

Group	A	B	C	D	Pooled Total
observations (N)	18	18	36	36	108
sum (Σx_i)	626.51	539.89	149.94	135.31	1,451.65
mean (x)	34.81	29.99	4.17	3.76	13.44
sum of squares (Σx^2_i)	32,252.15	24,563.57	1,293.46	907.17	59,016.35
sample variance (S^2)	614.46	492.36	19.11	11.39	369.20
sample standard deviation (S)	24.79	22.19	4.37	3.38	19.22
standard deviation of mean (SEx)	5.84	5.23	0.73	0.56	1.85

Table S2. Final statistical analysis of predicted and measured C_{trough} in the present patient population in relation to hepatic/renal adverse events

Source	sum of squares SS	degrees of freedom (νν)	mean square (MS)	F statistic	*p*-value
Group	19,620.99	3	6,540.33	34.21	1.78e-15
Error	19,883.54	104	191.19		
Total	39,504.53	107			

Table S3. Results of comparisons between groups in relation to hepatic/renal adverse events. The analyses have been performed by Bonferroni and Holm tests. Results are expressed as follows: [a], Bonferroni and Holm T-statistic; [b], Bonferroni p-value; [c], Holm p-value; [d]; Bonferroni and Holm interference

Groups	B	C	D
**A**	1.04[a] / 1.79[b]<br>0.60[c]<br>Not significant[d]	7.68 / 5.57e-11<br>4.65e-11<br>p<0.01	7.78 / 3.36e-11<br>3.36e-11<br>p<0.01
**B**		6.47 / 1.95e-08<br>9.77e-09<br>p<0.01	6.58 / 1.21e-08<br>8.05e-09<br>p<0.01
**C**			0.13 / 5.41<br>0.90<br>Not significant

Table S4. Post-hoc Tukey test in relation to hepatic/renal adverse events.

	Tukey HSD		
**Groups pair**	**Q statistic**	**p-value**	**Inferfence**
A vs B	1.48	0.70	Not significant
A vs C	10.86	0.001	** p<0.01
A vs D	11.00	0.001	** p<0.01
B vs C	9.15	0.001	** p<0.01
B vs D	9.30	0.001	** p<0.01
C vs D	0.18	0.90	Not significant

Note: **, statistically significant results

Table S5. Post-hoc Scheffé test in relation to hepatic/renal adverse events.

	Scheffé		
Groups pair	T-statistic	p-value	Inferfence
A vs B	1.04	0.78	Not significant
A vs C	7.68	3.63e-10	** $p<0.01$
A vs D	7.78	2.23e-10	** $p<0.01$
B vs C	6.47	1.03e-07	** $p<0.01$
B vs D	6.57	6.48e-08	** $p<0.01$
C vs D	0.13	1.00	Not significant

Note: **, statistically significant results

Table S6. Post-hoc Bonferroni and Holm tests in relation to hepatic/renal adverse events.

	Bonferroni and Holm	Bonferroni		Holm	
Groups pair	T-statistic	*p*-value	Inferfence	*p*-value	Inferfence
A vs B	1.04	1.79	insignificant	0.6294870	insignificant
A vs C	7.68	5.57e-11	** p<0.01	4.65e-11	** p<0.01
A vs D	7.78	3.36e-11	** p<0.01	3.36e-11	** p<0.01
B vs C	6.47	1.95e-08	** p<0.01	9.77e-09	** p<0.01
B vs D	6.57	1.21e-08	** p<0.01	8.05e-09	** p<0.01
C vs D	0.13	5.41	Not significant	0.90	Not significant

Note: **, statistically significant results

Table S7. Predicted and measured C_{trough} in the present patient population and statistical analysis showing significant differences among groups with and without hematological adverse events

Group	A	B	C	D	Pooled Total
observations (N)	16	16	38	38	108
sum (Σx_i)	559.36	487.13	217.10	188.07	1,451.66
mean (x)	34.96	30.45	5.71	4.95	13.44
sum of squares (Σx^2_i)	30,873.09	23,517.33	2,672.67	1,953.39	59,016.48
sample variance (S^2)	754.52	579.09	38.71	27.64	369.20
sample standard deviation (S)	27.47	24.06	6.22	5.26	19.22
standard deviation of mean (SEx)	6.87	6.02	1.01	0.85	1.85

Table S8. Final statistical analysis of predicted and measured C_{trough} in the present patient population in relation to hematological adverse events

Source	sum of squares SS	degrees of freedom (νν)	mean square (MS)	F statistic	*p*-value
Group	17,045.14	3	5,681.72	26.31	9.62e-13
Error	22,459.16	104	215.95		
Total	39,504.29	107			

Table S9. Results of comparisons between groups in relation to hematological adverse events

. The analyses have been performed by Bonferroni and Holm tests. Results are expressed as follows: [a], Bonferroni and Holm T-statistic; [b], Bonferroni p-value; [c], Holm p-value; [d]; Bonferroni and Holm interference

Groups	B	C	D
**A**	0.87[a] / 2.32[b]<br>0.77[c]<br>Not significant[d]	6.68 / 7.31e-09<br>6.09e-09<br>p<0.01	6.85 / 3.17e-09<br>3.17e-09<br>p<0.01
**B**		5.65 / 8.56e-07<br>4.28e-07<br>p<0.01	5.82 / 3.92e-07<br>2.61e-07<br>p<0.01
**C**			0.23/ 4.93<br>0.82<br>Not significant

Table S10. Post-hoc Tukey test in relation to hematological adverse events.

	**Tukey HSD**		
**Groups pair**	**Q statistic**	**p-value**	**Inferfence**
A vs B	1.23	0.80	Not significant
A vs C	9.44	0.001	** p<0.01
A vs D	9.69	0.001	** p<0.01
B vs C	7.99	0.001	** p<0.01
B vs D	8.23	0.001	** p<0.01
C vs D	0.32	0.90	Not significant

Note: **, statistically significant results

Table S11. Post-hoc Scheffé test in relation to hematological adverse events.

	Scheffé		
Groups pair	T-statistic	p-value	Inferfence
A vs B	0.87	0.86	Not significant
A vs C	6.68	4.01e-08	** p<0.01
A vs D	6.85	1.80e-08	** p<0.01
B vs C	5.65	3.73e-06	** p<0.01
B vs D	5.82	1.78e-06	** p<0.01
C vs D	0.23	1.00	Not significant

Note: **, statistically significant results

Table S12. Post-hoc Bonferroni and Holm tests in relation to hematological adverse events.

	Bonferroni and Holm	Bonferroni		Holm	
Groups pair	T-statistic	*p*-value	Inferfence	*p*-value	Inferfence
A vs B	0.87	2.32	insignificant	0.77	insignificant
A vs C	6.68	7.31e-09	** p<0.01	6.09e-09	** p<0.01
A vs D	6.83	3.17e-09	** p<0.01	3.17e-09	** p<0.01
B vs C	5.65	8.56e-07	** p<0.01	4.28e-07	** p<0.01
B vs D	5.82	3.92e-07	** p<0.01	2.61e-07	** p<0.01
C vs D	0.23	5.41	Not significant	0.82	Not significant

Note: **, statistically significant results

Figure S1

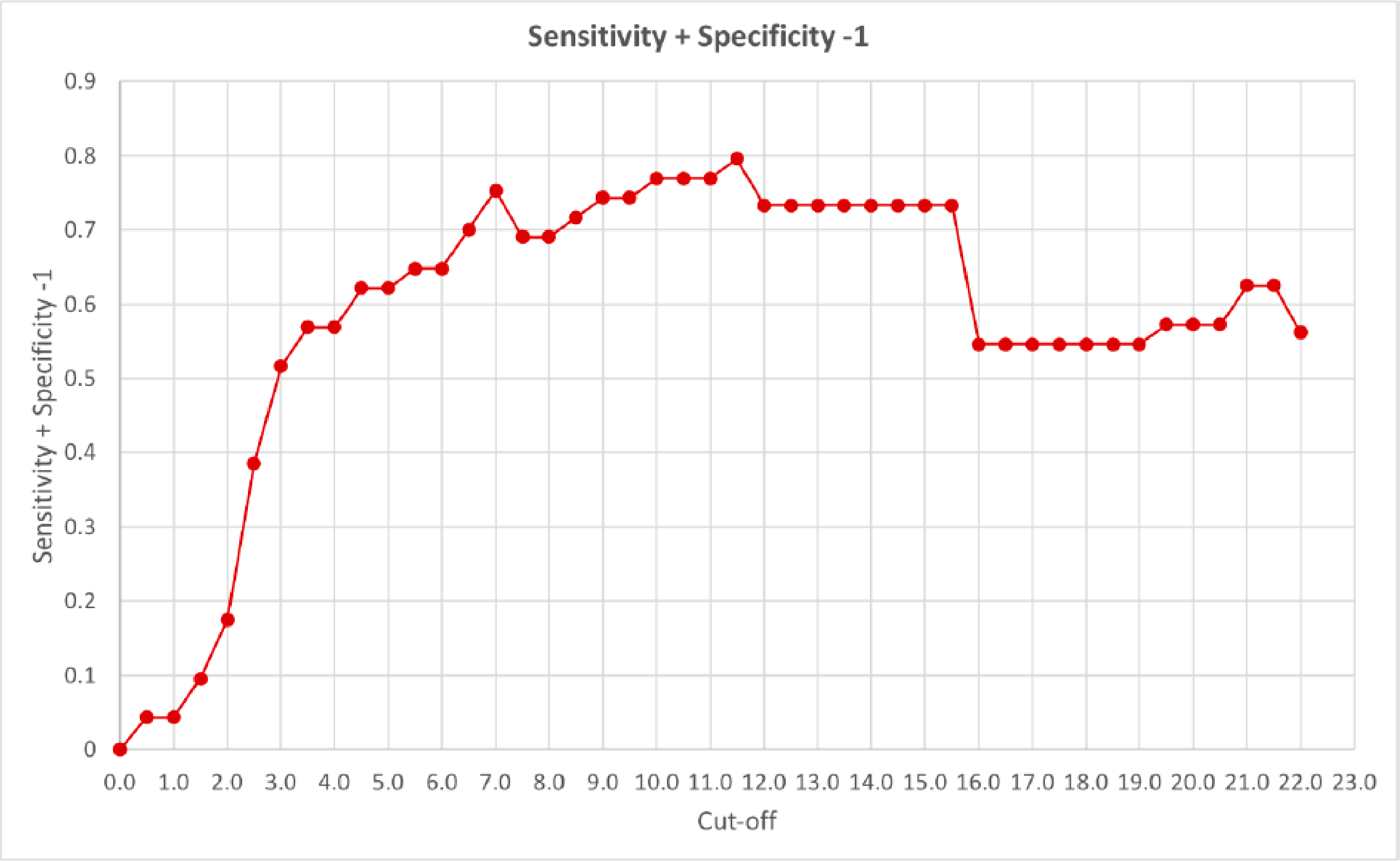

Figure S1. Graph plotting the Youden test results of Ctrough threshold for hematological toxicities.

Figure S2

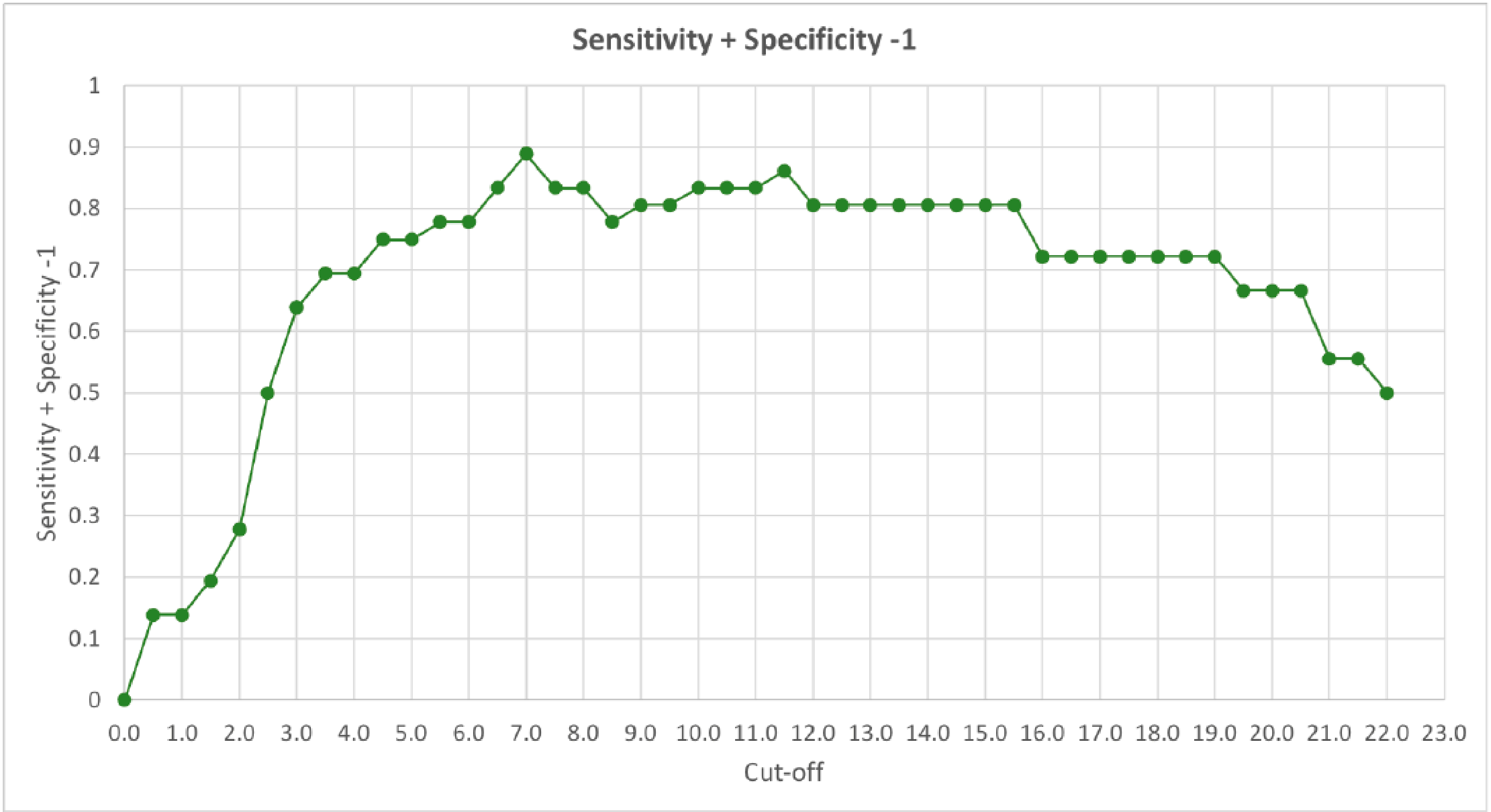

Figure S2. Graph plotting the Youden test results of C_{trough} threshold for hepatic/renal toxicities.
